# Supplementary material for: JAZ1-3 and MYC2-1 Synergistically Regulate the Transformation from Completely Mixed Flower Buds to Female Flower Buds in Castanea mollisima
Source: Int J Mol Sci. 2022 Jun 9;23(12):6452. doi: 10.3390/ijms23126452 (PMC9224291; doi:10.3390/ijms23126452)

Figure S1. KOG functional classification of transcriptome data of mixed flower buds of chestnut in pre-winter and post-winter.

a. Mixed flower buds of two kinds of chestnut in pre-winter.

b. Two mixed flower buds of chestnut in post-winter.

c. Fully mixed flower buds in pre-winter and post-winter.

d. Incomplete mixed flower buds in pre-winter and post-winter.

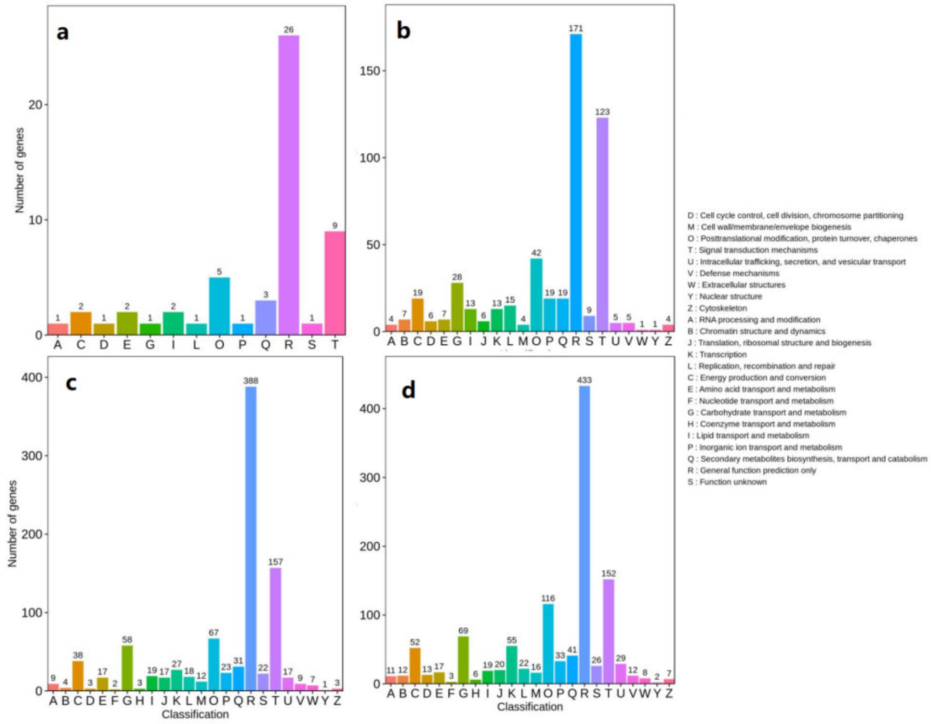

Figure S2. Go functional classification of transcriptome data of chestnut mixed flower buds in pre-winter

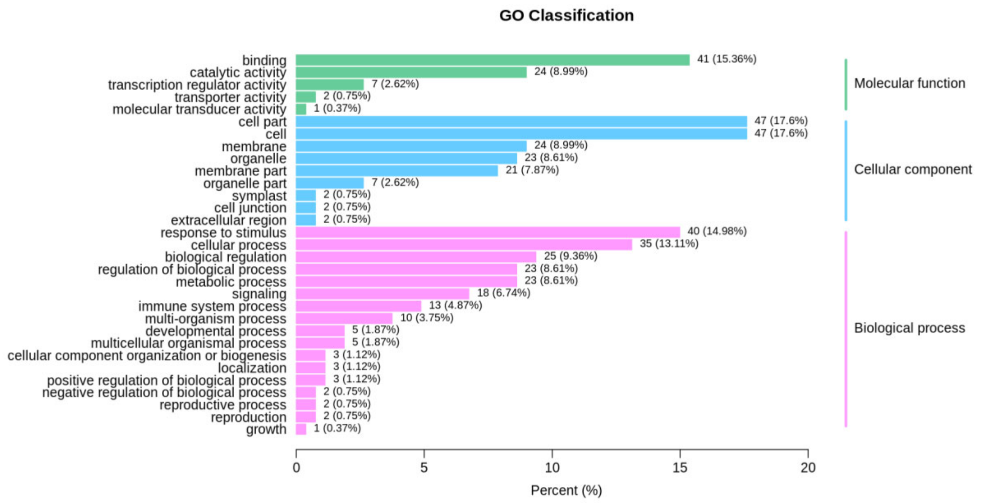

Figure S3. Go functional classification of transcriptome data of chestnut mixed flower buds in post-winter

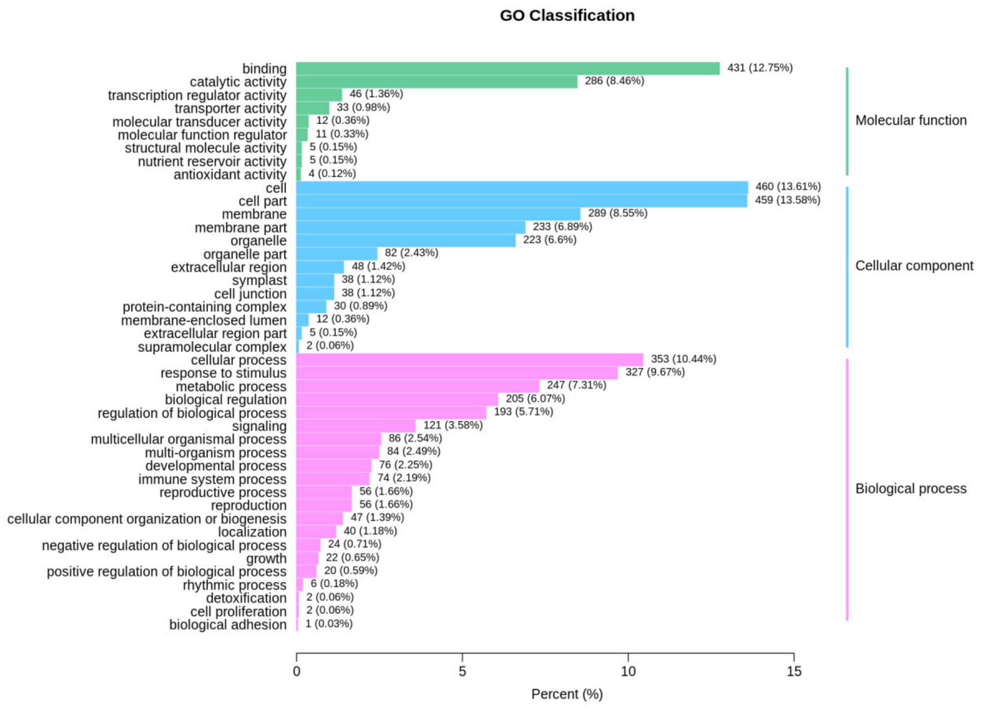

Figure S4. Go functional classification of completely mixed flower buds of *chestnut* in pre- and post-winter.

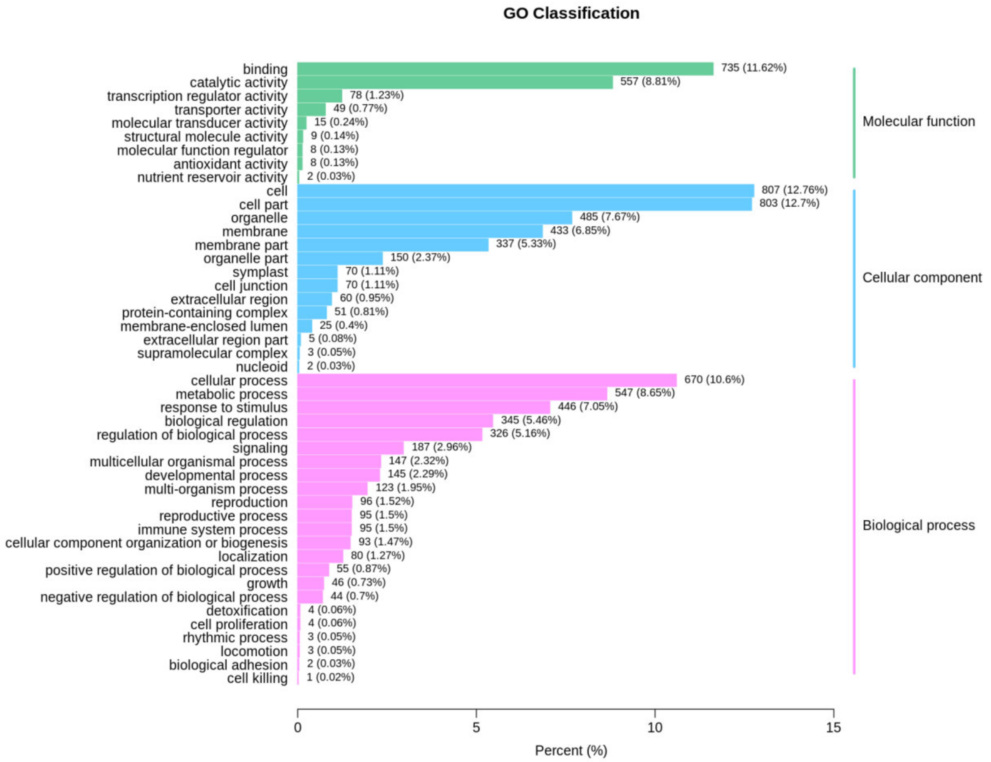

Figure S5. Go functional classification of incompletely mixed flower buds of *chestnut* in pre- and post-winter.

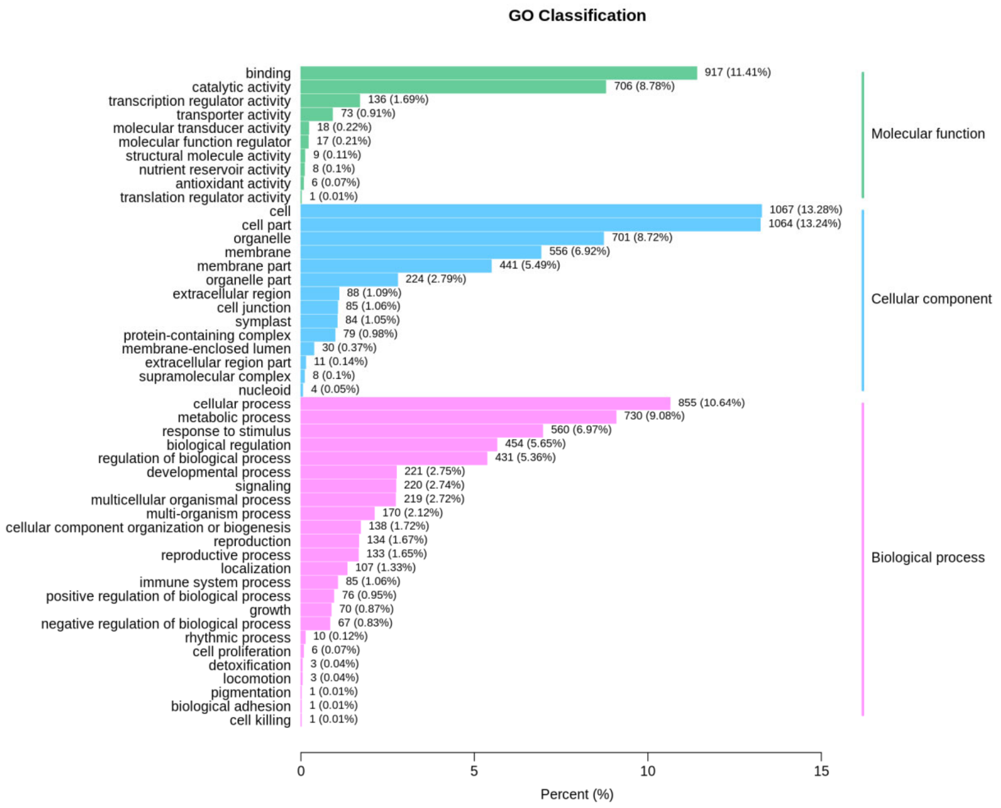

Figure S6. Volcanic map of differential metabolites. Each point in the figure corresponds to a metabolite. The abscissa is the logarithm of the quantitative difference multiple of a metabolite in two samples. The greater the absolute value, the greater the difference; The ordinate is the VIP value. The larger the value, the more significant the difference. Green points represent down regulation, red points represent up regulation, and gray points represent metabolites with no significant difference.

a. Two mixed flower buds in pre-winter.

b. Two mixed flower buds in post-winter.

c. Fully mixed flower buds in pre- and post-winter.

d. Incomplete mixed flower buds in pre- and post-winter winter.

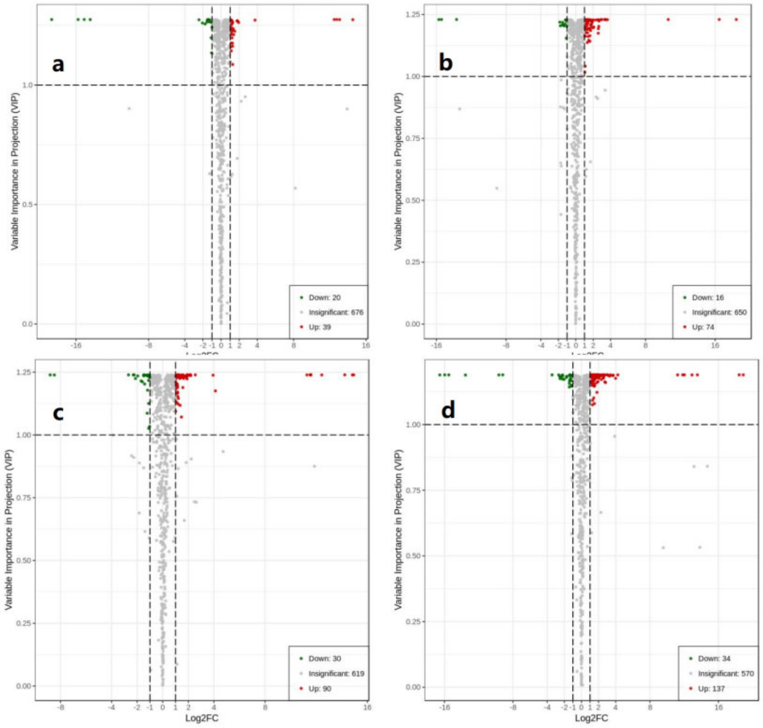

Figure S7. The heat map of differential metabolites in mixed flower buds of Chestnut. Each group of samples was divided into three groups of biological replicates, and each sample was repeated for three times. The results were analyzed from multiple perspectives by combining univariate statistical analysis and multivariate statistical analysis. The differential metabolites were screened by the combination of fold change and VIP value. The screening criteria were as follows: 1. The metabolites with fold change $\geqslant$ 2 and fold change $\leqslant$ 0.5 were selected; 2. On the basis of the above, the metabolites with VIP $\geqslant$ 1 were selected.

A1: Fully mixed flower buds in post-winter; B1: Incomplete mixed flower buds post-winter; A2: Fully mix flower buds pre-winter; B2: Incomplete mixed flower buds pre-winter.

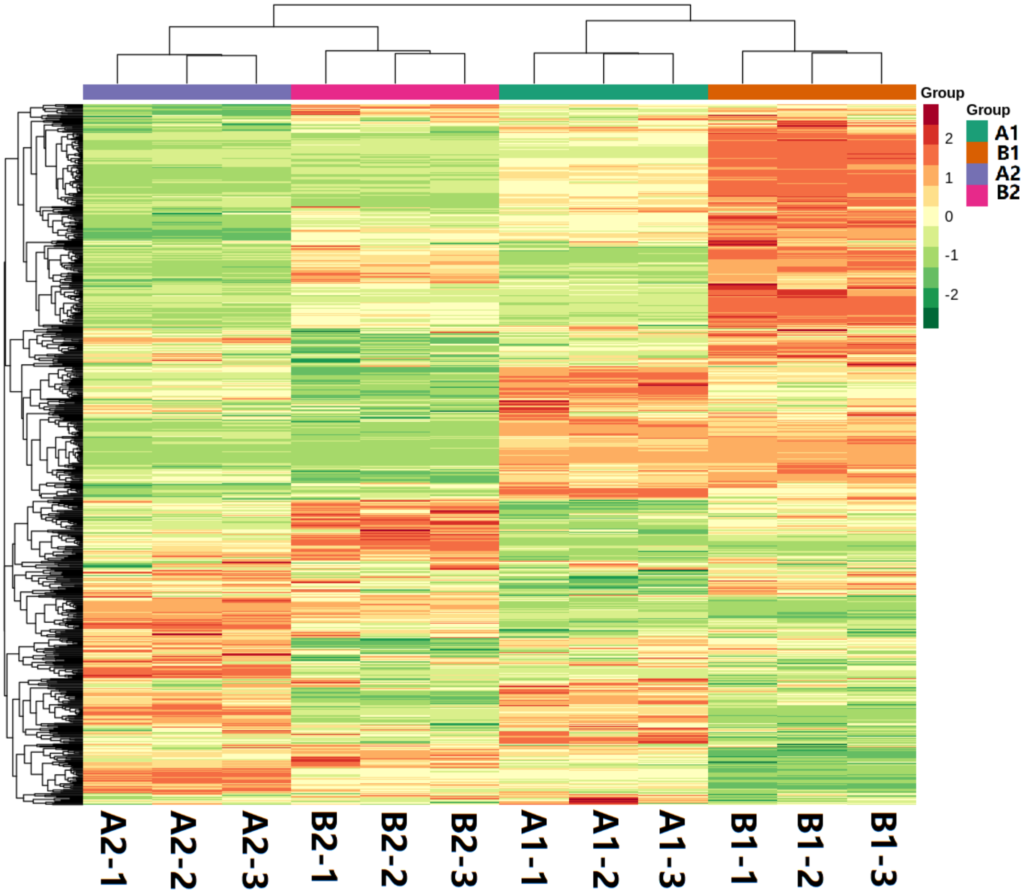

Figure S8. KEGG pathway analysis of differential metabolites in mixed flower buds of chestnut pre- and post-winter. Differential metabolites were annotated to KEGG database. According to the results of differential metabolites, the KEGG pathway is enriched and the bubble diagram is drawn. The rich factor is the ratio of the number of differentially expressed metabolites in the corresponding pathway to the total number of metabolites detected and annotated in the pathway. The smaller the value, the greater the enrichment degree. The closer pvalue is to 0, the more significant enrichment is. The size of the midpoint in the figure represents the number of significantly different metabolites enriched in the corresponding pathway.

a. Two mixed flower buds in pre-winter.

b. Two mixed flower buds in post-winter.

c. Fully mixed flower buds in pre- and post-winter.

d. Incomplete mixed flower buds in pre- and post-winter winter.

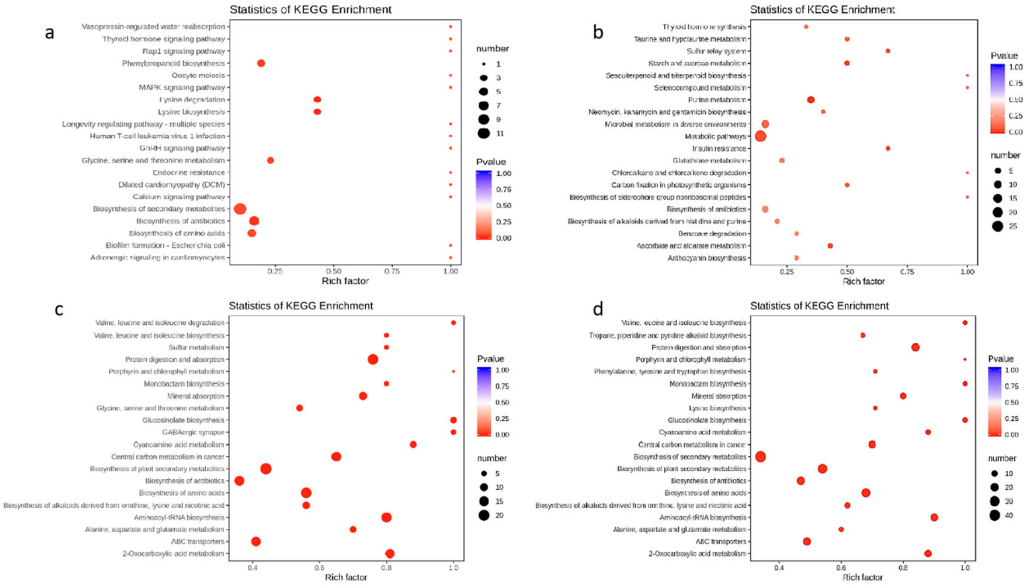

Supplement: Supplementary file 1 [file ijms-23-06452-s001.zip › 1 Supplementary figure.pdf]
