# Supplementary material for: JAZ1-3 and MYC2-1 Synergistically Regulate the Transformation from Completely Mixed Flower Buds to Female Flower Buds in Castanea mollisima
Source: Int J Mol Sci. 2022 Jun 9;23(12):6452. doi: 10.3390/ijms23126452 (PMC9224291; doi:10.3390/ijms23126452)
Supplement: Supplementary file 1 [file ijms-23-06452-s001.zip › 2 Supplementary table.pdf]

Table S1. Statistical table of sequencing data

Sample	Raw reads	Clean reads	Q20	Q30	GC content
A1-1	49,554,196	49,441,236	97.21%	92.51%	45.66%
A1-2	49,760,152	49,650,124	97.36%	92.77%	44.49%
A1-3	53,129,682	52,980,430	97.01%	92.05%	45.14%
A2-1	68,235,556	68,130,526	97.46%	92.90%	45.00%
A2-2	51,626,882	51,508,162	97.33%	92.64%	44.73%
A2-3	49,802,438	49,749,982	97.27%	92.54%	45.35%
B1-1	59,550,256	59,411,332	97.17%	92.41%	44.32%
B1-2	53,944,142	53,770,916	97.03%	92.14%	45.56%
B1-3	48,819,904	48,695,530	96.85%	91.78%	44.71%
B2-1	51,727,168	51,644,684	97.24%	92.45%	45.51%
B2-2	56,081,438	55,995,120	97.38%	92.79%	45.58%
B2-3	50,261,172	50,169,644	97.37%	92.76%	45.37%

Table S2. Clean reads comparison with reference genome

Sample	Unmapped reads	Mapped reads	Multiple mapped reads	mapQuality≥30(%)
A1-1	21.82%	78.18%	1.24%	75.86%
A1-2	15.85%	84.15%	1.32%	81.68%
A1-3	19.04%	80.96%	1.28%	78.58%
A2-1	19.31%	80.69%	1.45%	77.99%
A2-2	17.58%	82.42%	1.43%	79.77%
A2-3	22.20%	77.8%	1.33%	75.33%
B1-1	39.29%	60.71%	1.00%	58.86%
B1-2	25.62%	74.38%	1.10%	72.31%
B1-3	34.83%)	65.17%	0.97%	63.35%
B2-1	25.08%	74.92%	1.27%	72.57%
B2-2	25.71%	74.29%	1.24%	71.99%
B2-3	24.76%	75.24%	1.22%	72.99%

Note: Unmapped reads: There is no comparison of the number of reads on the genome; Mapped reads: Compare the number of reads in the last genome; Multiple mapped reads: The number of reads compared to multiple locations in the genome; map Quality >= 30: The following statistical items are based on the number of reads whose quality is not less than 30.

Table S3. Primers designed using Primer 5.0 software and used to real-time PCR analysis of GID1, GID2, DELLA1, DELLA2, MYC2-1, MYC2-2, MYC3, FT, LFY, SOC1, FLC, GA20ox1, GA3ox1 and SCF1 expression.

Gene name	Primer1	Primer2
Actin	TTGACTATGAGCAGGAACTT	TTGTAGGTGGTCTCGTGAAT
GID1	ACCTCAATGAATCCAAGAGG	ACAATCTTGGTAGTGCTCAAG
GID2	CGTGAGCTAGTAGAGAATGA	TCATCAAAGAACTCAAGCCT
DELLA1	TTCCGATACCGTTCTCTACA	AACTCTGTACATTCTGCTGC
DELLA2	GAGTGAAAGGTAGTGGTGAG	GTAGCAGTAGTAGTAGGAGGA

MYC2-1	ATCTCCTACAACACACCCTC	TTGAAACTCACAGTTCCCAT
MYC2-2	CTTTGCAGCAATTCGCATT	CATGCAACTTCACCAACTAC
MYC3	GGTGTGGAATGATGAGGAT	TGAGAATTTGCATAGCTTGG
FT	AGCACTACATTTCATCGTG	CTTTCACATGGTGCTGTGA
LFY	AGCAGCAGTATACTCGGT	CGTTGGTAGTGGTGTCAG
SOC1	GTGAGAGGAAAGACTCAGATG	GCATGTTCCTAGACCTTCTC
FLC	CGAAAATAGCGTTGCTCTTT	CTTGCTGTATACGAGTTCCA
GA20-1	ACCGATATGGGTAGTTTGACA	CGAGTATCGGTCCTTGGAAT
GA30-8	CTGTAATGCAATGGAAGTGTG	GCAATGATGAGTCTGTATGAGG
SCF1	GAGGAGGATGATGACGATG	AGGCTCTTCCTCATCGCTT

Table S4. Linear regression equation of various hormones

Hormones	Linear regression equation	Coefficient(R^2)
ABA	y = 28868.469x + 42055.7676	0.9988
cZ	y = 1261566.7899x + 25073.7856	0.9999
DZ	y = 808853.2338x - 1610845.7711	0.9982
H2JA	y = 97365.564x - 180296.8617	0.9995
IAA	y = 128094.5274x - 173184.0796	0.9982
IBA	y = 23985.7527x + 380416.6667	0.997
ICA	y = 506156.283x - 531119.3242	0.9936
IP	y = 1671470.4329x + 84041.4467	0.9994
JA	y = 61758.6022x + 890833.3333	0.9983
JA-ILE	y = 37317.7419x - 232500	0.9999
ME-IAA	y = 358913.0148x - 40086.4572	0.9993
MEJA	y = 99588.8465x - 52682.3579	0.9996
SA	y = 198185.4839x + 562500	0.999
tZ	y = 716880.9398x + 182271.6473	0.9999
ABA	y = 28868.469x + 42055.7676	0.9988
cZ	y = 1261566.7899x + 25073.7856	0.9999
DZ	y = 808853.2338x - 1610845.7711	0.9982
H2JA	y = 97365.564x - 180296.8617	0.9995
IAA	y = 128094.5274x - 173184.0796	0.9982
GA1	y = 33269.1542x + 1021.4428	0.9933
GA15	y = 191210.9375x - 57851.5625	0.9996
GA19	y = 42057.8125x + 14070.3125	0.9994
GA20	y = 116232.8125x + 27445.3125	0.9994
GA24	y = 109976.3441x + 2333333.3333	0.997
GA3	y = 90898.9247x + 2533333.3333	0.9968
GA4	y = 55518.2796x + 1823333.3333	0.9903
GA53	y = 40878.3465x - 6090.5512	0.9999
GA7	y = 640906.25x - 254843.75	0.9987
GA9	y = 24109.375x + 2484.375	0.9998

Table S5. Five hormone levels in different flower buds

Component	Group	Mean	SD	SE
IAA	A2	3.2667	0.0368	0.0212
IAA	A1	3.25	0.0498	0.0287
IAA	B2	3.2497	0.0115	0.0067
IAA	B1	3.2202	0.0125	0.0072
ME-IAA	A2	0.3247	0.0176	0.0102
ME-IAA	A1	0.344	0.0005	0.0003
ME-IAA	B2	0.3408	0.0015	0.0009
ME-IAA	B1	0.3437	0.0094	0.0054
ICA	A2	3.6062	0.0397	0.0229
ICA	A1	3.8114	0.0187	0.0108
ICA	B2	3.2788	0.0181	0.0105
ICA	B1	3.4368	0.0056	0.0032
IP	A2	0.4738	0.0094	0.0054
IP	A1	0.3131	0.0101	0.0058
IP	B2	0.5728	0.0077	0.0044
IP	B1	0.1187	0.0051	0.0029
tZ	A2	0.2561	0.0256	0.0148
tZ	A1	0.9487	0.0934	0.0539
tZ	B2	0.2182	0.0375	0.0216
tZ	B1	0.782	0.0192	0.0111
cZ	A2	0.1192	0.003	0.0017
cZ	A1	0.1279	0.0036	0.0021
cZ	B2	0.1773	0.0071	0.0041
cZ	B1	0.1843	0.0081	0.0047
DZ	A2	3.9801	0.0218	0.0126
DZ	A1	4.6086	0.0237	0.0137
DZ	B2	4.1027	0.0111	0.0064
DZ	B1	4.052	0.0276	0.016
MEJA	A2	111.2712	3.1177	1.8
MEJA	A1	1.936	0.0344	0.0199
MEJA	B2	1.6054	0.0257	0.0149
MEJA	B1	1.9521	0.024	0.0139
JA	A2	121.193	2.3204	1.3397
JA	A1	92.5858	0.7803	0.4505
JA	B2	135.3033	0.591	0.3412
JA	B1	76.0919	2.3786	1.3733
H2JA	A2	4.301	0.0358	0.0207
H2JA	A1	3.585	0.006	0.0035
H2JA	B2	3.6228	0.0387	0.0224
H2JA	B1	3.5569	0.0076	0.0044
JA-ILE	A2	140.9091	1.5021	0.8672
JA-ILE	A1	448.8522	21.233	12.2589

JA-ILE	B2	182.6802	2.4612	1.421
JA-ILE	B1	206.2527	3.2183	1.8581
SA	A2	238.5943	4.2762	2.4689
SA	A1	259.8331	5.0619	2.9225
SA	B2	327.8298	3.2336	1.8669
SA	B1	516.322	14.1466	8.1675
ABA	A2	50.7168	1.2259	0.7078
ABA	A1	17.8948	0.4057	0.2342
ABA	B2	32.15	0.8054	0.465
ABA	B1	18.7545	0.9024	0.521

Table S6, Gibberellin levels in different flower buds

Variable	Group	Value	SD	SE	CI
GA9	A2	1.9285	0.0302	0.0174	0.0749
GA9	A1	1.4717	0.0710	0.0410	0.1764
GA9	B2	1.3832	0.0810	0.0468	0.2012
GA9	B1	1.8879	0.0915	0.0528	0.2274
GA19	A2	4.2377	0.1613	0.0931	0.4007
GA19	A1	1.0967	0.0375	0.0216	0.0931
GA19	B2	2.2640	0.0815	0.0470	0.2024
GA19	B1	0.7291	0.0155	0.0089	0.0384
GA20	A2	0.6854	0.0292	0.0169	0.0727
GA20	A1	0.2858	0.0230	0.0133	0.0572
GA20	B2	0.6042	0.0083	0.0048	0.0205
GA20	B1	0.4740	0.0232	0.0134	0.0576
GA53	A2	0.3425	0.0041	0.0024	0.0103
GA53	A1	0.2522	0.0037	0.0021	0.0091
GA53	B2	0.3186	0.0024	0.0014	0.0059
GA53	B1	0.3151	0.0022	0.0013	0.0054

Table S7. Sampling time and number of chestnut flower buds

Sample	Sapling time	Sampling location
A2-1	Dec 17th 2017	The second bud from top to bottom
A2-2	Dec 17th 2017	The second bud from top to bottom
A2-3	Dec 17th 2017	The second bud from top to bottom
A1-1	Mar 15th 2018	The second bud from top to bottom
A1-2	Mar 15th 2018	The second bud from top to bottom
A1-3	Mar 15th 2018	The second bud from top to bottom
B2-1	Dec 17th 2017	The second bud from bottom to top
B2-2	Dec 17th 2017	The second bud from bottom to top
B2-3	Dec 17th 2017	The second bud from bottom to top
B1-1	Mar 15th 2018	The second bud from bottom to top
B1-2	Mar 15th 2018	The second bud from bottom to top
B1-3	Mar 15th 2018	The second bud from bottom to top

Table S8. Primers designed using Primer 5.0 software and used to vector construction primer.

Gene name	Primer1	Primer2
CmJAZ1-3y1	ggccatggagATGGCGAGCTCGTCGGAGTT	acgtcgacCTACTGCTTTAGCTGAGCAGCCA
CmMYC2-2y1	ctgaattcATGTCACATTCTGCTCATTCCAT	caggatccCTTGGTCCTTTTACCTTTAAGCC
CmJAZ1-3d1	ggtctagaATGGCGAGCTCGTCGGAGTT	taggatccCTACTGCTTTAGCTGAGCAGCCA
CmMYC2-2d1	ggtctagaATGTCACATTCTGCTCATTCCAT	taggatccCTTGGTCCTTTTACCTTTAAGCC
CmFT527d1	atctcgagACGATGATTTAGGATGCTGGG	caaagcttCCCTATACACAATAACAACTTGGA
CmFT933d1	atctcgagCAACGTAATGATTGAATGCGA	ctaagcttCCCTATACACAATAACAACTTGGA
CmLFY2-465d1	atctcgagTGTCCCATCCACTCTTCCT	ctaagcttAGCCTCGTAAGCAAATGCCA
CmLFY2-674d1	atctcgagCCTTCTATTTATCAGTCCCTCCT	ctaagcttAGCCTCGTAAGCAAATGCCA
CmLFY3-473d1	atctcgagTTCGACTTATCGGTCAACCTAG	ctaagcttAGACACTGACCTCAAGTATAGAA
CmLFY3-878d1	atctcgagTTTGATCGTGTATTTGAAGGC	ctaagcttAGACACTGACCTCAAGTATAGAA
